# Regioselective Hydroxylation of Phloretin, a Bioactive Compound from Apples, by Human Cytochrome P450 Enzymes

**DOI:** 10.3390/ph13110330

**Published:** 2020-10-22

**Authors:** Ngoc Anh Nguyen, Ngoc Tan Cao, Thi Huong Ha Nguyen, Thien-Kim Le, Gun Su Cha, Soo-Keun Choi, Jae-Gu Pan, Soo-Jin Yeom, Hyung-Sik Kang, Chul-Ho Yun

**Affiliations:** 1 School of Biological Sciences and Biotechnology, Graduate School, Chonnam National University, Yongbong-ro 77, Gwangju 61186, Korea; ngocanh61093@gmail.com (N.A.N.); caongoctan93@gmail.com (N.T.C.); huongha0207@gmail.com (T.H.H.N.); thienkim.1611@gmail.com (T.-K.L.); soojin258@chonnam.ac.kr (S.-J.Y.); kanghs@jnu.ac.kr (H.-S.K.); 2 Namhae Garlic Research Institute, 2465-8 Namhaedaero, Gyeongsangnamdo 52430, Korea; gscha450@gmail.com; 3 Korea Research Institute of Bioscience and Biotechnology, 125 Gwahak-ro, Daejeon 34141, Korea; sookeun@kribb.re.kr (S.-K.C.); jgpan@kribb.re.kr (J.-G.P.); 4 School of Biological Sciences and Technology, Chonnam National University, Yongbong-ro 77, Gwangju 61186, Korea

**Keywords:** human cytochrome P450, human liver microsomes, human metabolite, phloretin, polyphenol, regioselective hydroxylation

## Abstract

Phloretin, the major polyphenol compound in apples and apple products, is interesting because it shows beneficial effects on human health. It is mainly found as a form of glucoside, phlorizin. However, the metabolic pathway of phloretin in humans has not been reported. Therefore, identifying phloretin metabolites made in human liver microsomes and the human cytochrome P450 (P450) enzymes to make them is interesting. In this study, the roles of human liver P450s for phloretin oxidation were examined using human liver microsomes and recombinant human liver P450s. One major metabolite of phloretin in human liver microsomes was 3-OH phloretin, which is the same product of a bacterial CYP102A1-catalyzed reaction of phloretin. CYP3A4 and CYP2C19 showed *k*_cat_ values of 3.1 and 5.8 min^−1^, respectively. However, CYP3A4 has a 3.3-fold lower *K*_m_ value than CYP2C19. The catalytic efficiency of a CYP3A4-catalyzed reaction is 1.8-fold higher than a reaction catalyzed by CYP2C19. Whole-cell biotransformation with CYP3A4 was achieved 0.16 mM h^−1^ productivity for 3-OH phlorein from 8 mM phloretin at optimal condition. Phloretin was a potent inhibitor of CYP3A4-catalyzed testosterone 6β-hydroxylation activity. Antibodies against CYP3A4 inhibited up to 90% of the microsomal activity of phloretin 3-hydroxylation. The immunoinhibition effect of anti-2C19 is much lower than that of anti-CYP3A4. Thus, CYP3A4 majorly contributes to the human liver microsomal phloretin 3-hydroxylation, and CYP2C19 has a minor role.

## 1. Introduction

**Figure 1:**
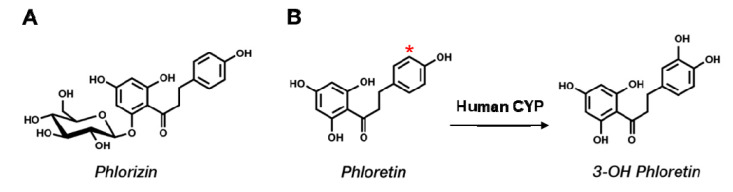
Chemical structures of phlorizin, phloretin, and 3-OH phloretin. (**A**) Phlorizin is a major glucoside form of phloretin. (**B**) Phloretin’s conversion to its product is catalyzed by human cytochrome P450 (P450) in the presence of reduced β-nicotinamide adenine dinucleotide phosphate (NADPH). A star (*) marks the hydroxylation site on phloretin catalyzed by P450.

Phloretin [3-(4-hydroxyphenyl)-1-(2,4,6-trihydroxyphenyl)propan-1-one] is a natural polyphenol compound found mainly in most parts of apple trees, including in apple skin and apple pomace [1] (Figure 1). It is a dihydrochalcone among flavonoids and is mostly found as the glucoside phlorizin in plants [2]. Similar to many polyphenol compounds, phloretin displays antioxidant properties and interferes with the growth of several types of cancer cells [3]. Recently, we found that bacterial CYP102A1 regioselectively hydroxylates phloretin to produce 3-OH phloretin and that 3-OH phloretin dramatically inhibits differentiating 3T3-L1 preadipocytes into adipocytes and lipid accumulation [4]. Phloretin and phlorizin are currently accepted as health-beneficial polyphenols from apples useful in treating hyperglycemia [5]. 

Phlorizin (phloretin 2’-O-glucose) is the major form of phloretin found in apples. Phlorizin is absorbed in the small intestine and transported via sodium–glucose transporters (SGLTs) with subsequent deglycosylation by β-glucosidase activity of lactate-phlorizin hydrolase [6,7]. Phlorizin is known to be a specific competitive inhibitor of SGLTs in intestines (SGLT-1) and kidneys (SGLT-2), and it seems to show beneficial effects on obesity and diabetes [8]. The major cytochrome P450 (P450 or CYP) enzyme involved in the metabolic reactions of flavonoids is CYP1A2, whereas other human liver P450s (such as CYP3A4, CYP2D6, CYP2E1, CYP2C19, and CYP2E1) contributed as minor roles to flavonoid metabolism when the metabolisms of 30 flavonoid aglycones were screened using human liver P450 enzymes [9]. The main oxidation reaction is hydroxylation at the carbon in the phenyl ring. However, the metabolic pathway of phloretin in humans has not been reported. 

Phloretin shows a potent chemopreventive effect against aflatoxin B1 via its inhibitory effect on CYP3A4 and CYP1A2. On the other hand, it has an inductive effect on glutathione S-transferase activity [10]. Phloretin can also inhibit the catalytic activity of human CYP1A1 [11]. Phloretin’s inhibitory effect on P450-catalyzed reactions may suggest that phloretin can be a substrate to human P450 enzymes. However, to the best of our knowledge, no reports exist on phloretin metabolism in humans.

This study aimed to determine major phloretin metabolites in the human liver and find the contributing roles of particular P450 enzyme(s) involved in forming the metabolites. For these aims, we performed catalytic activity assays of phloretin 3-hydroxylation with human liver microsomes (HLMs) and recombinant enzymes. We found 3-OH phloretin as the major metabolite in HLMs, and that CYP3A4 contributes as a major enzyme to produce 3-OH phloretin. Such information can have a considerable clinical impact regarding potential drug–drug and food–drug interactions and interindividual variations of drug metabolism.

## 2. Results

### 2.1. Phloretin Metabolism by Human Liver Microsomes and Identification of the Major Metabolite 

**Figure 2:**
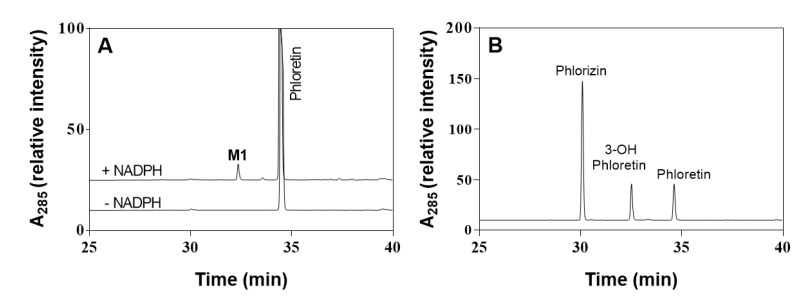
HPLC chromatograms of phloretin and its metabolites via human liver microsomes. (**A**) Peaks of the reaction mixtures were identified by comparing their retention times (*t*_R_) with those of standard compounds: (**B**) 3-OH phloretin (*t*_R_ = 32.4 min), phloretin (*t*_R_ = 34.6 min), and phlorizin (*t*_R_ = 30.1 min).

**Figure 3:**
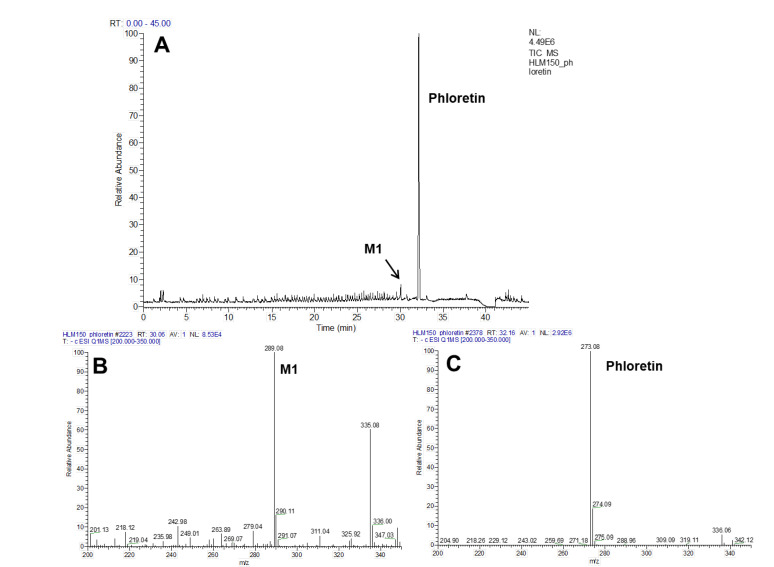
LC-MS analyses of the phloretin metabolite via human liver microsomes (HLMs). LC-MS chromatogram of phloretin metabolites catalyzed by HLMs in the presence (**A**) of NADPH. The MS spectra show that the protonated molecular ions of 3-OH phloretin (**B**) and phloretin (**C**) were 289 and 273, respectively.

First, to determine HLMs’ ability to oxidize phloretin, the catalytic activity of HLMs toward phloretin was measured via a 200-μM substrate for 60 min at 37 °C. Figure 2A shows the high-performance liquid chromatography (HPLC) profiles of phloretin and its metabolites via HLMs. One major metabolite appeared at a retention time of 32.4 min. The major product of HLMs in the presence NADPH showed the same retention time of 3-OH phloretin, which is phloretin’s major product produced by CYP102A1 enzymes [4]. The turnover number for 3-OH phloretin formation was 0.052 min^−1^ under this experimental condition (Figure 2). To confirm the product’s chemical structure, we did an analysis via liquid chromatography-mass spectrometry (LC-MS) (Figure 3 and Figure S1). A full-scan chromatogram of the reaction of a mixture of phloretin with HLMs appears in Figure 3. When the mixture was compared with the matrix control, phloretin shows a major metabolite—M1. Accuracy results for molecular weight and an elemental analysis indicated that M1 (*m/z* 289) was a monohydroxylated metabolite when compared to the substrate of phloretin (*m/z* 273). This result indicates that P450s in HLMs catalyze phloretin’s regioselective hydroxylation to produce a catechol product. A minor metabolite (*t*_R_ = 33.5 min) was also observed (Figure 2A). However, we could not characterize this compound because any apparent mass data with LC-MS analysis were not observed.

Next, we examined each human P450’s possible contributions to the phloretin 3-hydroxylation. We examined the catalytic activity of several human CYPs, such as CYP3A4, CYP1B1, CYP1A2, CYP2D6, CYP2A6, CYP2E1, and CYP2C19. Only CYP2C19 and CYP3A4 have apparent phloretin 3-hydroxylation activity. The same product, 3-OH phloretin, was detected in phloretin metabolized via CYP2C19 and CYP3A4 in the presence of NADPH (Figure S2). To identify phloretin metabolites generated by CYP2C19 and CYP3A4, analyses of HPLC (Figure S2) and LC-MS were done (Figures S3 and S4). The mass spectrum of phloretin showed a protonated molecular ion ([M-H]^+^) at *m/z* 273. The metabolites had *m/z* 289, so these results indicate that all HLMs, CYP3A4, and CYP2C19 samples produce only one major product, a 3-OH product.

### 2.2. Kinetics Parameters and Total Turnover Numbers (TTNs) of Phloretin Hydroxylation via HLMs and Recombinant Human CYPs 

Table 1 and Figure S5 show the steady-state kinetics of 3-OH phloretin formation via HLMs and the two recombinants of CYP3A4 and CYP2C19. HLMs had a lower *k*_cat_ value (0.094 min^−1^) and an intermediate *K*_m_ value (120 μM) compared to CYP3A4 and CYP2C19. Although CYP2C19 had the highest *k*_cat_ value of 5.8 min^−1^, it showed the highest *K*_m_ value of 208 μM. Although CYP3A4 had a lower *k*_cat_ value of 3.1 min^−1^ compared to CYP2C19, it showed a much lower *K*_m_ value of 63 μM. Therefore, the CYP3A4-catalyzed reaction’s efficiency was 1.8-fold higher than that of CYP2C19. This result suggests CYP3A4 is the major contributor toward the production of 3-OH phloretin in human livers. 

**Figure 4:**
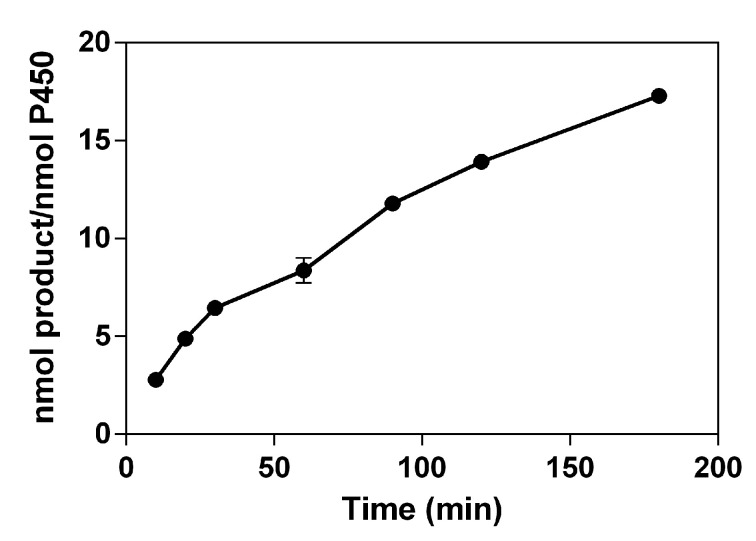
Time profiles of 3-OH phloretin formation via HLMs. Total turnover numbers (TTNs) for HLMs were determined via HPLC after accomplishing the reactions at indicated times at 37 °C. The 3-OH phloretin was measured in the reaction mixtures containing P450 (0.40 μM of HLMs), an NADPH regeneration system, and phloretin (0.50 mM) for a total volume of 0.25 mL.

When the time profile of 3-OH phloretin formation by HLMs ranged from 10 to 180 min, the formation of 3-OH phloretin gradually increased with the reaction time. The total turnover number (TTN, nmol product/nmol enzyme) was 17.3 at 180 min into the reaction (Figure 4). 

It is well known that cytochrome *b*_5_ (*b*_5_) plays an important role in liver P450-derived substrate metabolism. The effect of *b*_5_ on the phloretin 3-hydroxylation activity of CYP3A4 and CYP2C19 was examined. The *b*_5_ increased the activities of CYP3A4 and CYP2C19 by 52% and 41%, respectively (Figure S6). It is known that the stimulatory effects of *b*_5_ on both enzymes are very diverse [12].

### 2.3. Inhibition of Phloretin Hydroxylation Activity by Antibodies in HLMs 

**Figure 5:**
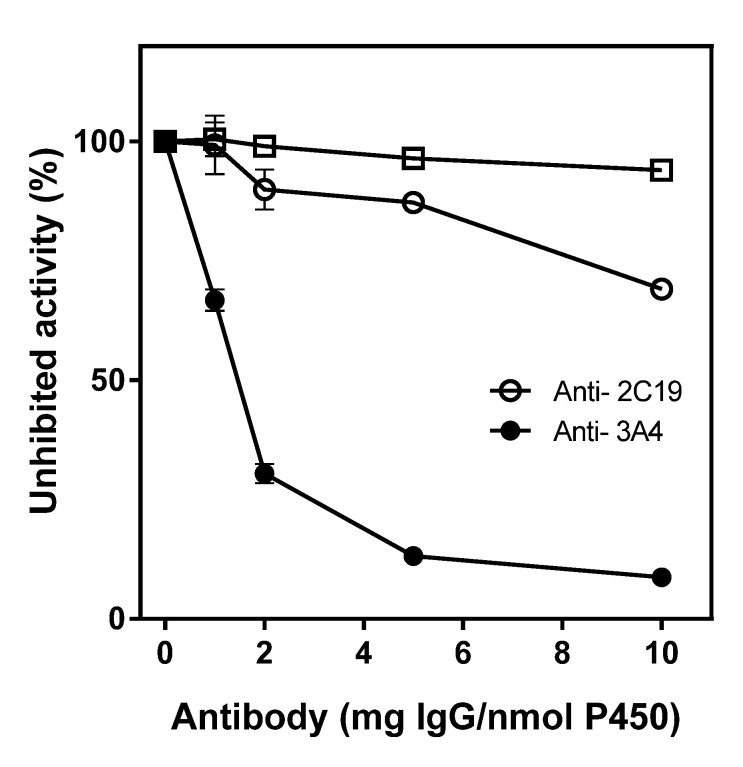
Immunoinhibition of antibodies on phloretin 3- hydroxylation activity catalyzed by HLMs. The effects of anti-CYP3A4 (●), anti-CYP2C19 (○), and preimmune IgG (□) on phloretin 3- hydroxylation activity in HLMs were measured. Values represent the means ± SD of three determinations.

To determine to what extent CYP2C19 and CYP3A4 contribute to phloretin hydroxylation, immunoinhibition studies with anti-CYP2C19 and anti-CYP3A4 were conducted. Anti-CYP2C19 and anti-CYP3A4 inhibited phloretin hydroxylation activity as the antibodies’ concentration increased (Figure 5). Anti-CYP2C19 inhibited 10% and 13% of the phloretin hydroxylation activity when 2 and 5 mg of Immunoglobulin G (IgG) per nmol of P450 were added, respectively. Anti-CYP3A4 showed a much higher inhibitory effect of 70% and 87% when 2 and 5 mg of IgG per nmol of P450 were added, respectively. This result indicates that CYP3A4 and CYP2C19 are a major and a minor CYP for phloretin 3-hydroxylation reaction, respectively. These immunoinhibition studies also suggest that CYP3A4 and CYP2C19 cause almost all the phloretin oxidation in HLMs. 

### 2.4. Inhibitory Effect of Phloretin on Testosterone 6β-Hydroxylation Catalyzed by CYP3A4

CYP3A4 was the major P450 for phloretin 3-hydroxylation activity in HLMs, so we examined phloretin’s effect on the CYP3A4 catalytic activity using testosterone as a substrate. Testosterone 6β-hydroxylation is a widely used marker of CYP3A4’s enzyme activity [13]. 

**Figure 6:**
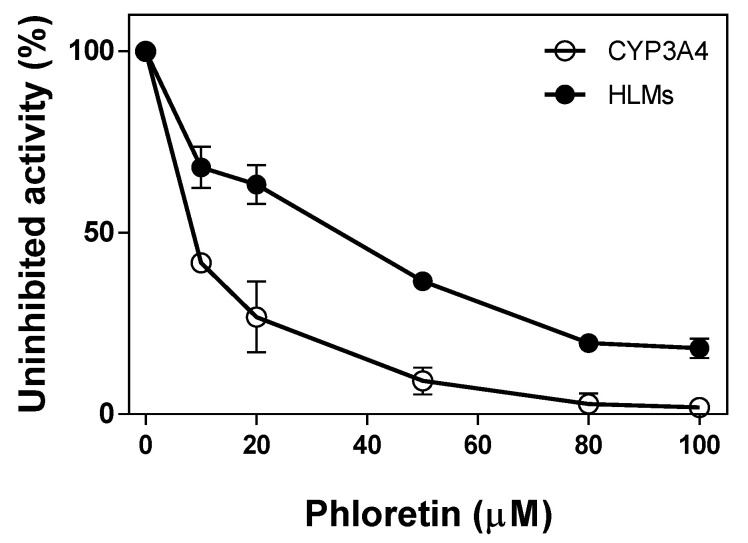
Phloretin’s inhibitory effect on the testosterone 6β-hydroxylation by CYP3A4 and HLMs. After preincubating phloretin with CYP3A4 (○) and HLMs (●) in the presence of NADPH for 5 min, testosterone (0.50 mM) was added to start the reaction. The reactions were terminated after 30 min. The formation of 6β-OH testosterone was measured by HPLC. Values represent the means ± SD of three determinations.

Phloretin inhibited testosterone 6β-hydroxylation catalyzed by CYP3A4 and HLMs as the concentration of phloretin increases (Figure 6). It showed a high inhibitory effect on CYP3A4-catalyzed testosterone 6β-hydroxylation. When 10 μM and 50 μM of phloretin were preincubated with CYP3A4, 40% and 91% of the CYP3A4 activity was inhibited, respectively. Phloretin’s inhibitory effect on the activity of HLMs was much lower than on CYP3A4. When 50 μM of phloretin was preincubated with HLMs, 63% of activity was inhibited. The half maximal inhibitory concentration (IC_50_) values of testosterone 6β-hydroxylation via phloretin against CYP3A4 and HLMs were 13 μM and 92 μM, respectively. Thus, phloretin can be a substrate of CYP3A4 with a high affinity, and other P450(s) in addition to CYP3A4 may be involved in testosterone 6β-hydroxylation in HLMs. It is known that human CYP2C9 and CYP2C19 have the catalytic activity of testosterone 6β-hydroxylation in addition to CYP3A4 [14]. 

### 2.5. Whole-Cell Biotransformation of Phloretin 

In this study, we developed CYP3A4 expressing whole-cell biocatalyst to produce 3-OH phloretin for industrial applications. To find the best host *Escherichia coli* cells of recombinant human CYP3A4 for whole-cell biotransformation, six different types of competent *E. coli* cells (Shuffle T7, Rosetta, MG1655, DH5α-F’IQ, BL21, and JM109) were tested for CYP3A4 expression in a Luria–Bertani medium [15]. The optimum temperature and culture time were determined by measuring enzyme activity from 20 °C to 30 °C for a set of culture times of 10, 20, 22, 24, and 26  h. Results show DH5α-F’IQ was the best strain for CYP3A4 expression after a 22 h culture at 30 °C. The expression level of CYP3A4 was 0.20 μM. Thus, the DH5α-F’IQ strain was selected for the whole-cell biotransformation experiments. 

**Figure 7:**
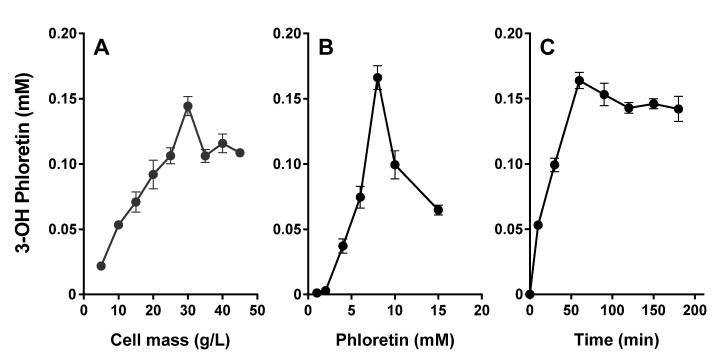
Whole-cell biotransformation of phloretin by a recombinant *E. coli* cell expressing CYP3A4. (**A**) Effect of cell concentration on the conversion of phloretin into 3-OH phloretin by whole cells expressing CYP3A4. The reactions were performed with cell concentrations from 5 to 45 g L^−1^ and 10 mM of phloretin at 37 °C for 60 min. (**B**) The effect of phloretin concentration on the production of 3-OH phloretin by recombinant whole cells expressing CYP3A4. The reactions were performed in a range from 1 to 15 mM with 30 g L^−1^ cells at 37 °C for 60 min. (**C**) Time courses of phloretin into 3-OH phloretin by recombinant *E. coli* cells expressing CYP3A4. The reactions were performed with 30 g L^−1^ cells and 8 mM of phloretin at 37 °C for 180 min. Data are shown as the means ± the SD of triplicate determinations.

Production of 3-OH phloretin, the major metabolite, from the substrate phloretin was optimized by varying the cell mass and the substrate concentrations (Figure 7A,B). Production of 3-OH phloretin was increased up to 30 g cell L^−1^ (Figure 7A) and 8 mM phloretin (Figure 7B). However, above this concentration, the production plateaued. These results indicate that the optimal concentrations of CYP3A4 harboring DH5α-F’IQ cells and phloretin were 30 g of cell L^−1^ and 8 mM of phloretin, respectively. The production of 3-OH phloretin increased when substrate concentrations increase up to 8 mM. *E. coli* cells expressing the CYP3A4 produced 0.16 mM of 3-OH phloretin after 60 min reaction, showing 0.16 mM h^−1^ productivity when 8 mM of phloretin was used. 

Finally, we demonstrated that high biotransformation of 8 mM phloretin into 3-OH phloretin was achieved by 30 g L^−1^ CYP3A4 expressing whole cells at 37 °C for 180 min (Figure 7C). The 3-OH phloretin production gradually increased with increasing time up to 60 min when it was saturated. The productivity for 60 min reaction with CYP3A4 whole-cell bioconversion was 0.16 mM h^−1^. These results provide CYP3A4 expressing whole-cells, which are useful key biocatalysts for 3-OH phloretin production. In addition, high cell density culture and whole-cell immobilization of these whole-cell biocatalysts should be further studied for industrial application.

## 3. Discussion

Consuming polyphenol-rich foods are associated with health benefits, especially regarding chronic diseases, such as cancer and obesity [16]. It is generally accepted that apples have diverse beneficial effects on blood pressure, lipids, vascular function, hyperglycemia, inflammation, and even cancer. The cardioprotective effect of apples may be associated with polyphenols [17,18]. A well-known bioactive polyphenol in apples is phloretin, which is mainly found as a form of glucoside: phlorizin [19,20]. Phloretin is a main compound, which belongs to the dihydrochalcone class among flavonoids and also exists in strawberries and pears. Phloretin has a wide range of biological effects, including antioxidation, anti-inflammatory, and anti-tumor properties, and it reduces vascular endothelial dysfunction and liver injury [21,22,23]. At present, phloretin and its glucoside phlorizin are generally considered health-beneficial polyphenols from apples [5]. In this study, we found that 3-OH phloretin was a major metabolite in HLMs. Recently, we found that 3-OH phloretin, a monohydroxylated product of phloretin catalyzed by bacterial CYP102A1, inhibits the differentiation of 3T3-L1 preadipocytes into adipocytes. We also reported that lipid accumulation was dramatically inhibited by 3-OH phloretin but increased by phloretin. Furthermore, after oral administration of *Lithocarpus polystachyus* Rehd to in vivo rat models, which has several flavonoids including phlorizin, 3-OH phloretin, and its conjugates with sulfate were identified in plasma, urine, and feces [24]. This result suggests that 3-OH phloretin, a human metabolite of phloretin, may show higher beneficial effects than those of phloretin itself. Further studies with 3-OH phloretin are necessary to find its effects on human health. 

Human P450s have a dominant contribution to metabolizing clinical drugs and other foreign chemicals, including dietary natural polyphenols from fruits and vegetables [25,26]. CYP3A4 and CYP2C19 are the principal catalysts for metabolizing phloretin into 3-OH phloretin. CYP3A4 is most abundant in livers and small intestines, and it contributes to the metabolism of over 50% of clinical medicines [27]. CYP3A4 shows a very broad substrate specificity, and it can also metabolize diverse molecules with different sizes and chemical structures via diverse oxygenation reactions [28,29,30]. CYP2C19 also plays an important role in processing or metabolizing important therapeutic drugs. In particular, the CYP2C19 gene is highly polymorphic [31]. Humans have a wide interindividual variation in the expression of individual P450s [17]. Understanding human P450s, which participate in the oxidation of phloretin, would help in assessing individual susceptibility to this potent inhibitor against adipocyte differentiation and lipid accumulation [4]. Formation of 3-OH phloretin by P450 enzymes in the liver with the consumption of apples and apple products might cause the inhibition of adipocyte differentiation and lipid accumulation. Increasing apple intake may provide health benefits and potentially help weight loss.

P450 enzymes are the major contributing enzymes involved in the oxidation reactions of dietary natural compounds obtained from food and fruits in humans [25,26]. With the same approach to the issue of MIST (Metabolites in Safety Testing) for drugs [32], efficient production systems are required to permit the large-scale production of human metabolites of dietary compounds obtained from food and fruits. As chemical synthesis methods sometimes cannot synthesize these metabolites, biocatalytic production of the metabolites using whole-cell biotransformation with a recombinant expression of human P450s would be a promising approach to provide the metabolites for the assessment of efficacy and safety of each dietary compound.

Polyphenol compounds are abundant in the human diet, including in vegetables and fruits [33]. In this study, we found that CYP3A4 was the major enzyme to metabolize phloretin in the liver and that phloretin inhibited the CYP3A4 catalytic activity. This result suggests a potent interaction of phloretin, a popular polyphenol in apples, with clinical drugs metabolized by CYP3A4. As CYP3A4 is also essential to metabolize a large set of clinical drugs, phloretin treatment could affect the usage of many clinical drugs or cause significant side effects. Popular dietary supplements and foods also have a high risk for interacting with drugs metabolized by CYP3A4, the most popular drug-metabolizing enzyme in human livers and small intestines. However, the potential effects of drugs’ sensitivity to phloretin remain unknown.

## 4. Materials and Methods 

### 4.1. Materials

Phloretin, the oxidized form of β-nicotinamide adenine dinucleotide phosphate (NADP^+^), glucose-6-phosphate, glucose-6-phosphate dehydrogenase from *Leuconostoc mesenteroides*, acetonitrile, methanol, and ethyl acetate were purchased from Sigma–Aldrich (St. Louis, MO, USA). Other chemicals used in this work were obtained with the highest grade commercially available, and they were used without further purification.

Recombinant human P450s were heterologously expressed in *E. coli* with a pCW vector containing human P450 cDNA (CYP3A4 or CYP2C19) and rat NADPH-P450 reductase (CPR) [34,35]. pCW vectors expressing P450 and CPR were constructed in previous studies: CYP1A2, CYP3A4, CYP2C19, CYP1B1, CYP2E1, CYP2D6, and CYP2A6 [34,35]. Membrane fractions expressing P450 and CPR were prepared, as described previously [34,35], and were used for the catalytic activity assays.

HLMs (a human liver microsome pool) were purchased from ThermoFisher Scientific (Walthan, MA, USA). 

Antibodies against CYP3A4 and 2C19 were prepared in the previous work [36].

### 4.2. Oxidation of Phloretin Catalyzed by Human Liver Microsomes

Human liver microsomal reactions included incubation at 37 °C in 0.25-mL incubation mixtures containing P450 (0.4 μM) in a potassium phosphate buffer (100 mM, pH 7.4), an NADPH-generating system (NGS, final concentration: 10 mM glucose-6-phosphate, 0.50 mM NADP^+^, and 1.0 IU yeast glucose-6-phosphate dehydrogenase per mL), and the reaction substrate (0.50 mM). Incubations were performed generally for 60 min with or without the NADPH-generating system (NGS). Phloretin and its metabolites were extracted twice with ethyl acetate, followed by centrifugation (1000× g, 20 min) to separate the organic and aqueous layers [37,38].

After the organic layer was concentrated using a stream of nitrogen gas, the residue was dissolved in 180 µL of mobile A:B (6:4, *v*/*v*). Mobile phase A contained 0.5% (*v*/*v*) methanol, 0.1% (*v*/*v*) formic acid, and 99.4% water. Mobile phase B was 100% acetonitrile. Product formation was analyzed by HPLC, and samples (30 µL) were injected onto a Gemini C18 column (4.6 mm × 150 nm, 5 µm; Phenomenex, Torrance, CA, USA). Increasing mobile phase B from 9 to 100% (*v*/*v*) over 37.5 min was performed as follows: initial 11 min, 9% acetonitrile; 11–13 min, 15% acetonitrile; 13–20 min, 17% acetonitrile; 20–37 min, 60% acetonitrile; and 37–37.5 min, 100% acetonitrile with the flow rate of 1.0 mL. The stepwise gradient was done using a gradient pump (LC-20AD; Shimadzu, Kyoto, Japan). The detection of phloretin and its metabolites was performed at 285 nm. 

The kinetic parameters for substrate oxidation via recombinant P450s and HLMs were estimated using 0.40 μM of recombinant P450 or HLMs, an NGS and phloretin (10–1000 µM) in a potassium phosphate buffer (100 mM, pH 7.4). The reaction was initiated by the addition of a NADPH regeneration system and continued at 37 °C. Reaction times for recombinant P450a and HLMs were 10 min and 30 min, respectively. Quantifying the metabolites was performed by comparing peak areas of the metabolites to the mean peak areas of the 3-OH phloretin standard compound, which was prepared as previously described [4].

The kinetic parameters (*K*_m_ and *k*_cat_) were calculated via the nonlinear weakening study using GraphPad Prism software (GraphPad, Software Inc., San Diego, CA, USA). The equation was applied for Michaelis–Menten kinetics. 

The effects of *b*_5_ on phloretin 3-hydroxylation catalyzed by CYP3A4 and CYP2C19 were performed by incubating 0.40 μM of recombinant P450, 0.80 μM of recombinant NADPH-P450 reductase, 0.8 μM of recombinant human *b*_5_, an NGS, and phloretin (300 µM) in a potassium phosphate buffer (100 mM, pH 7.4). The reaction was started by adding an NGS and continued at 37 °C for 10 min [39]. 

### 4.3. Phloretin’s Inhibitory Effect on Testosterone 6β-Hydroxylation Catalyzed via HLMs and CYP3A4

To examine phloretin’s effect on testosterone 6β-hydroxylation catalyzed via CYP3A4 and HLMs, reaction mixtures were performed at 37 °C in 0.25 mL incubation mixtures containing 0.40 μM of P450 in a potassium phosphate buffer (100 mM, pH 7.4) and an NGS. After preincubation with indicated phloretin concentrations (10–100 μM) for 5 min in the presence of NGS at 37 °C, testosterone (0.50 mM) was added to start the reaction. After reacting for 30 min, the products were stopped and extracted via ethyl acetate, followed by centrifugation (1000× *g*, 20 min) to separate the organic and aqueous layers. 6β-OH testosterone was analyzed via HPLC. The organic layer was obtained and then concentrated using a stream of nitrogen gas, and the residue of the organic layer was dissolved in 180 µL of mobile phase (60% methanol). Product formation was analyzed by HPLC, and samples (30 μL) were injected into a Gemini C18 column (4.6 mm × 150 nm, 5 µm; Phenomenex, Torrance, CA). The flow rate was 1.0 mL using 60% methanol as a mobile phase, and the substrate and its products were detected at 285 nm.

### 4.4. Immunoinhibition of Antibodies on Phloretin 3-Hydroxylation

Inhibition studies using specific antibodies to CYP3A4 and CYP2C19 were performed by incubating HLMs with varying concentrations of anti-P450 IgG for 30 min at 23 °C before adding other components, including phloretin and an NGS, required for catalytic activity [40]. The control experiments with varying concentrations of the preimmune IgG were done.

### 4.5. LC-Mass Spectrometric Analysis of a Phloretin Metabolite

To investigate the major product’s mass, a CYP3A4 reaction mixture containing 0.40 µM of P450, 500 µM of phloretin, and NGS was used in 0.25 mL of a potassium phosphate buffer (100 mM, pH 7.4). After these reaction mixtures were incubated at 37 °C for 30 min, injection samples were prepared as described above. Analyses of the mass values of phloretin and its products were performed using a TSQ Quantum™ Access MAX Triple Quadrupole Mass Spectrometer on an Accela 1250 HPLC system (ThermoFisher Scientific, Waltham, MA, USA). The samples were separated on a ZORBAX SB-C18 column (250 mm × 4.6 mm i.d. 5 µm; Agilent, Santa Clara, CA, USA) at a flow rate of 1 mL/min. The mobile phases were (A) 0.1% (*v*/*v*) formic acid in water and (B) acetonitrile. The isocratic flow of the mobile phase was (A) 60% and (B) 40% on HPLC. The injection volume was 10 µL. The mass spectra were recorded via electrospray ionization in positive mode to characterize phloretin metabolites. The collision energy and scan rate were 10 V and 0.5 spectra/s, respectively.

### 4.6. Optimal Expression of CYP3A4 in E. coli

Six different strains of competent *E. coli* cells (BL21, DH5α-F’IQ, MG1655, Shuffle T7, Rosetta, and JM109) harboring the CYP3A4 plasmid were inoculated into Luria-Bertani (LB) broth supplemented with ampicillin (100 µg/mL). The strain was grown at 37 °C with shaking at 180 rpm overnight. It was then inoculated to the Terrific Broth (TB) medium and was grown to absorbance at 600 nm (OD_600_) ~0.6–0.8. After that, isopropyl–β-D-thiogalactopyranoside (0.5 mM) and δ-aminolevulinic acid (1.0 mM) were added for enzyme induction and functional expression. After the cultures were grown at 20, 25, and 30 °C with shaking at 170 rpm, Fe^2+^·CO versus Fe^2+^ difference (CO) spectra were measured at indicated cultured times from 10 h up to 26 h. The P450 concentrations were quantified via the CO-difference spectra measurement using an extinction molecular coefficient of ε = 91 mM/cm [41].

### 4.7. Whole-Cell Biotransformations 

*E. coli* DH5αF’-IQ cells expressing recombinant human CYP3A4 were obtained at the stationary phase and were used for biotransformation experiments. The cells were collected by centrifugation at 6000× g and 4 °C for 10 min. After the removal of the supernatant, the cell pellet was resuspended in a potassium phosphate buffer (100 mM, pH 7.4) containing 6.0 mM of magnesium acetate and 10 mM of dextrose. The optimal concentrations of *E. coli* cells and phloretin substrate for 3-OH phloretin production were determined via *E. coli* cells ranging from 5 to 45 g per liter with 8.0 mM of phloretin and via phloretin ranging from 1.0 to 15 mM with 30 g cells per liter for 1 h, respectively. Production of 3-OH phloretin via recombinant *E. coli* cells expressing CYP3A4 was performed with a potassium phosphate buffer (100 mM, pH 7.4) containing 30 g of *E. coli* cells per liter and 8.0 mM of phloretin at 37 °C for 60 min. The formation rate of 3-OH phloretin was quantified by HPLC, as described above.

## 5. Conclusions

In this study, the roles of human liver P450s in phloretin oxidation were investigated as phloretin is a popular polyphenol compound that is rich in apples. The major metabolite of phloretin is 3-OH phloretin. We found that CYP3A4 was a major P450 and CYP2C19 was a minor P450 to catalyze phloretin 3-hydroxylation in the human liver by using recombinant human P450 enzymes and HLMs. Phloretin was a potent inhibitor of CYP3A4-catalyzed testosterone 6β-hydroxylation activity. As CYP3A4 is the most popular drug-metabolizing enzyme in the human liver, possible interactions of phloretin with clinical drugs metabolized by CYP3A4 should be considered to avoid any drug side effects with apple consumption. 

## Figures and Tables

**Table 1:** Kinetic parameters of phloretin hydroxylation via human liver microsomes (HLMs), CYP3A4, and CYP 2C19.

Enzymes	*k*_cat_ (min^−1^ )	*K*_m_ (μM)	*k*_cat_/*K*_m_ (min^−1^μM^−1^)
HLMs	0.094 ± 0.006	120 ± 36	0.00083 ± 0.00025
CYP3A4	3.1 ± 2.9	63 ± 11	0.049 ± 0.023
CYP2C19	5.8 ± 0.6	208 ± 50	0.028 ± 0.007

## Supplementary Materials

The following are available online at https://www.mdpi.com/1424-8247/13/11/330/s1: Figure S1. LC-MS analyses of the phloretin metabolite by HLMs; Figure S2. HPLC chromatograms of phloretin and its metabolites via HLMs, CYP3A4 and CYP2C19LC-MS analyses of the phloretin metabolite by CYP3A4; Figure S3. LC-MS analyses of the phloretin metabolite by CYP3A4; Figure S4. LC-MS analyses of the phloretin metabolite by CYP2C19; Figure S5. The kinetic parameters for 3-OH phloretin formation by human CYP3A4, CYP2C19, and HLMs; Figure S6. Effects of cytochrome *b*_5_ on phloretin 3-hydroxylation by CYP3A4 and CYP2C19.
