# Supplementary material for: Regioselective Hydroxylation of Phloretin, a Bioactive Compound from Apples, by Human Cytochrome P450 Enzymes
Source: Pharmaceuticals (Basel). 2020 Oct 22;13(11):330. doi: 10.3390/ph13110330 (PMC7690628; doi:10.3390/ph13110330)
Supplement: Supplementary file 1 [file pharmaceuticals-13-00330-s001.pdf]

pharmaceuticals MDPI

Supporting Information

Regioselective Hydroxylation of Phloretin, a Bioactive Compound from Apples, by Human Cytochrome P450 Enzymes

Ngoc Anh Nguyen [1], Ngoc Tan Cao [1], Thi Huong Ha Nguyen [1], Thien –Kim Le [1], Gun Su Cha [2], Soo-Keun Choi [3], Jae-Gu Pan [3], Soo-Jin Yeom [1,4], Hyung-Sik Kang [1,4] and Chul-Ho Yun [1,4,*]

[1] School of Biological Sciences and Biotechnology, Graduate School, Chonnam National University, Yongbong-ro 77, Gwangju 61186, Republic of Korea; ngocanh61093@gmail.com (N.A.N.); caongoctan93@gmail.com (N.T.C.); huongha0207@gmail.com (T.H.H.N.); thienkim.1611@gmail.com (T.-K.L.)

[2] Namhae Garlic Research Institute, 2465-8 Namhaedaero, Gyeongsangnamdo 52430, Republic of Korea; gscha450@gmail.com (G. S.C.)

[3] Korea Research Institute of Bioscience and Biotechnology, 125 Gwahak-ro, Daejeon, 34141, Republic of Korea; sookeun@kribb.re.kr (S.-K.C.); jgpan@kribb.re.kr (J.-G.P.)

[4] School of Biological Sciences and Technology, Chonnam National University, Yongbong-ro 77, Gwangju 61186, Republic of Korea; soojin258@chonnam.ac.kr (S.-J.Y.); kanghs@jnu.ac.kr (H.-S.K.)

* Correspondence: chyun@jnu.ac.kr (C.-H.Y); Tel. +82-62-530-2194

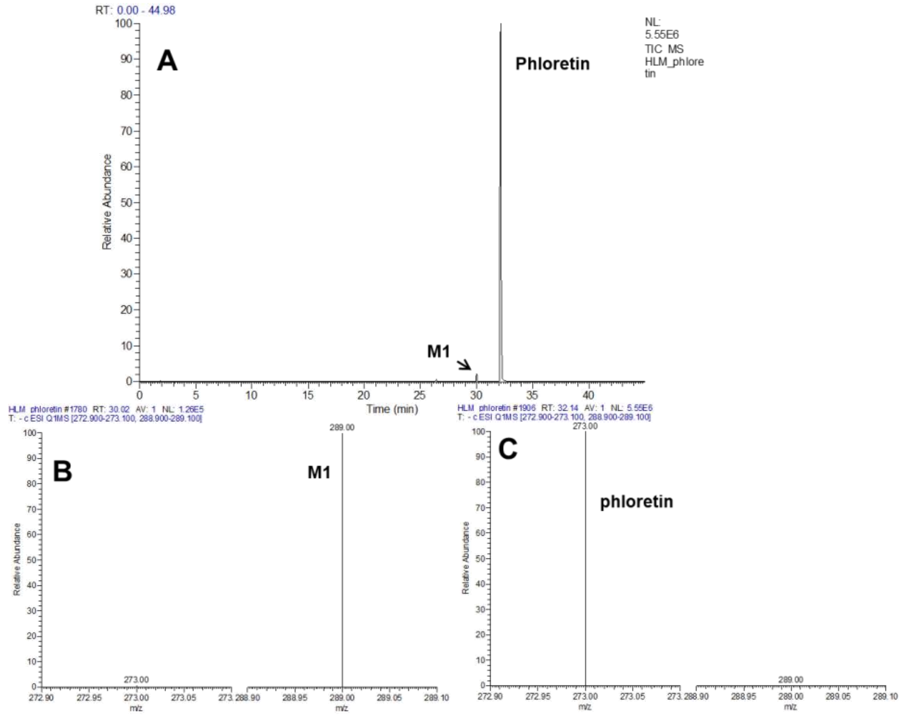

Figure S1. LC-MS analyses of the phloretin metabolite by HLMs. LC-MS chromatogram of phloretin metabolites catalyzed by HLMs in the presence (**A**) of NADPH. The MS spectra with SIM mode demonstrate that the protonated molecular ions of 3-OH phloretin (B) and phloretin (C) were 289 and 273, respectively.

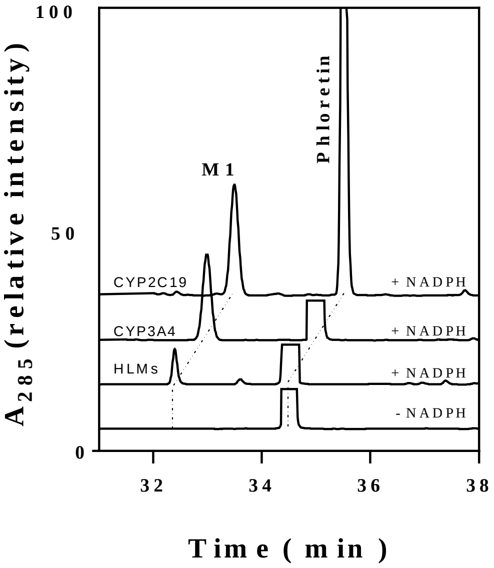

Figure S2. HPLC chromatograms of phloretin and its metabolites via HLMs, CYP3A4 and CYP2C19. Peaks of the reaction mixtures of HPLC traces were identified by comparing their retention times with those of 3-OH phloretin (t_R = 32.4 min) and phloretin (t_R = 34.6 min).

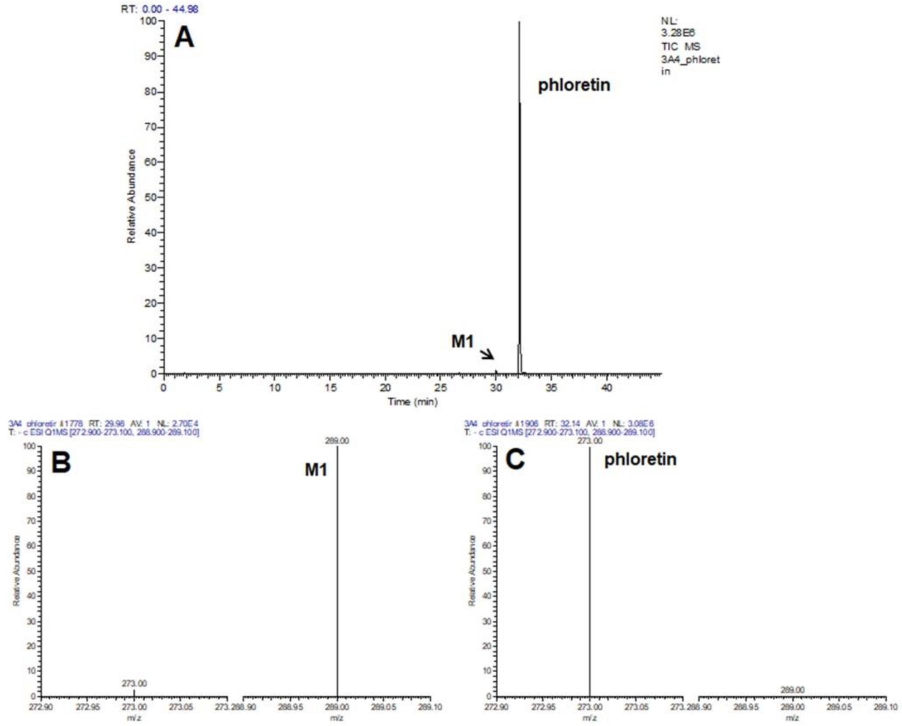

Figure S3. LC-MS analyses of the phloretin metabolite by CYP3A4. LC-MS chromatogram of phloretin metabolites catalyzed by CYP3A4 in the presence (**A**) of NADPH. The MS spectra with SIM mode demonstrate that the protonated molecular ions of 3-OH phloretin (B) and phloretin (C) were 289 and 273, respectively.

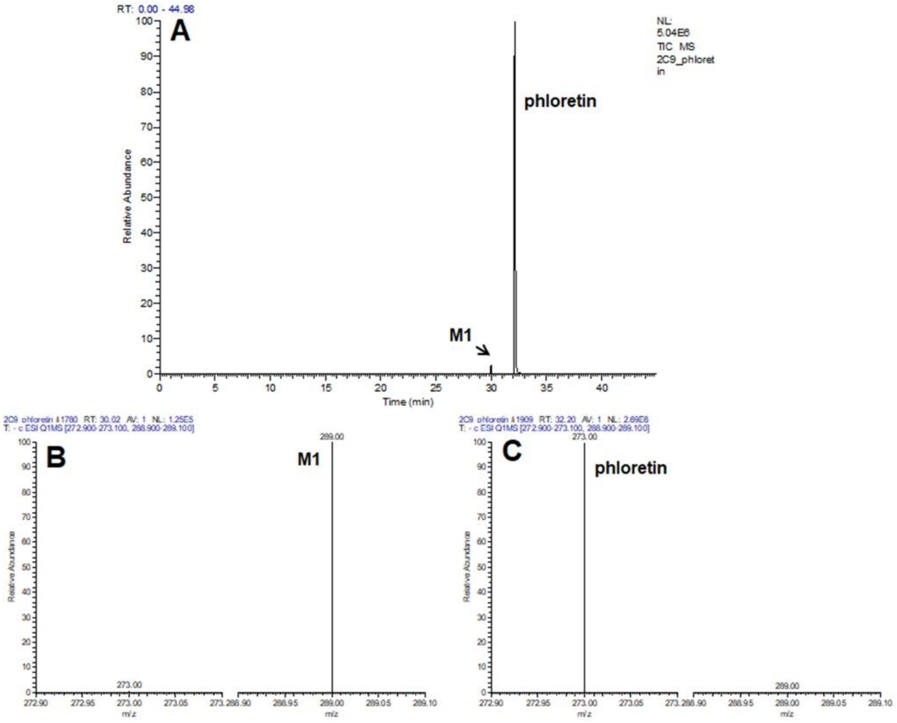

Figure S4. LC-MS analyses of the phloretin metabolite by CYP2C19. LC-MS chromatogram of phloretin metabolites catalyzed by CYP2C19 in the presence (**A**) of NADPH. The MS spectra with SIM mode demonstrate that the protonated molecular ions of 3-OH phloretin (B) and phloretin (C) were 289 and 273, respectively.

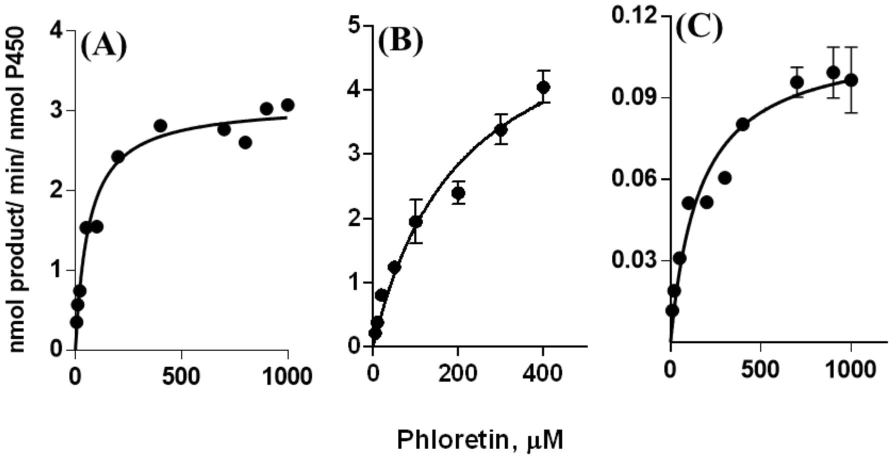

Figure S5. The kinetic parameters for 3-OH phloretin formation by human CYP3A4 (A), CYP2C19 (B), and HLMs (C). Reaction time and P450 concentrations of all incubation in these experiments were within the linear range of metabolite formation. The substrate concentrations for phloretin hydroxylation were in the ranges of 10-1000 μM for CYP3A4 (A), 10-400 μM for CYP2C19 (B), 10-1000 μM for HLMs (C), respectively.

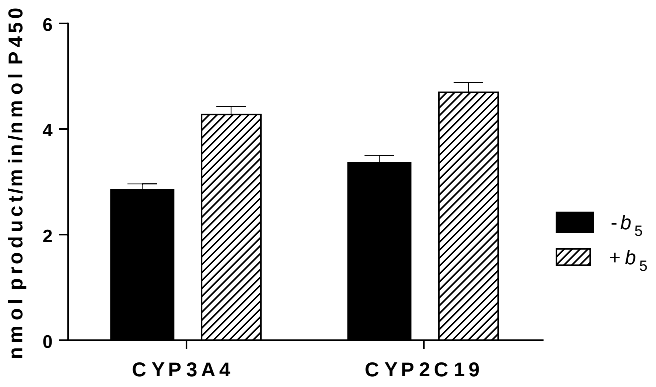

Figure S6. Effects of cytochrome b_5 on phloretin 3-hydroxylation by CYP3A4 and CYP2C19. The experimental conditions are described under Materials and Methods. Values represent the means ± SD of three determinations.
